# The DREB7 transcription factor enhances salt tolerance in soybean plants under salt stress

**DOI:** 10.1515/biol-2025-1153

**Published:** 2025-08-20

**Authors:** Giang Thu Nguyen, Yen Thi Hai Nguyen, Hung Duc Nguyen, Mau Hoang Chu, Quan Huu Nguyen

**Affiliations:** Department of Biology, Thai Nguyen University of Education, Thai Nguyen 24000, Vietnam; Department of Foundation Medicine, Thai Nguyen University of Medicine and Pharmacy, Thai Nguyen 24000, Vietnam; Department of Biotechnology, Thai Nguyen University of Sciences, Thai Nguyen 24000, Vietnam; Department of Fundamental Sciences and Basic Medical Sciences, Pham Ngoc Thach University of Medicine, Ho Chi Minh City 740310, Vietnam

**Keywords:** *In silico* molecular docking, *Glycine max*, *GmDREB7* gene, *GmP5CS* gene, salt stress, proline accumulation

## Abstract

DREB7 in *Glycine max* (L) is a novel trans-acting transcription factor (TF) that binds to the *cis*-acting sequences of promoters to activate the expression of downstream genes in response to abiotic factors. This study presents the experimental results and analyzes the relationship between the overexpression of the *GmDREB7* and *GmP5CS*, as well as the proline content, in transgenic soybean lines. The results of qRT-PCR analysis of four TG1 transgenic soybean lines (TG1-2, TG1-5, TG1-7, and TG1-10) showed that the *GmDREB7* gene had significantly higher transcriptional expression under untreated and salt stress conditions. Under salt stress conditions, the two transgenic lines, TG1-5 and TG1-10, had the most significant increase in *GmDREB7* and *GmP5CS* gene expression levels, as well as the highest proline accumulation (*P* < 0.05). The *in silico* molecular docking analysis confirmed a specific interaction between the DREB7 protein and GmP5CS promoter. These findings demonstrate that overexpression of the gene encoding the TF DREB7 enhanced the transcription of the *GmP5CS* gene and increased proline accumulation in soybean plants under salt stress conditions. The *GmDREB7* gene can be a promising candidate for enhancing salt tolerance in soybeans.

## Graphical abstract

**Figure:**
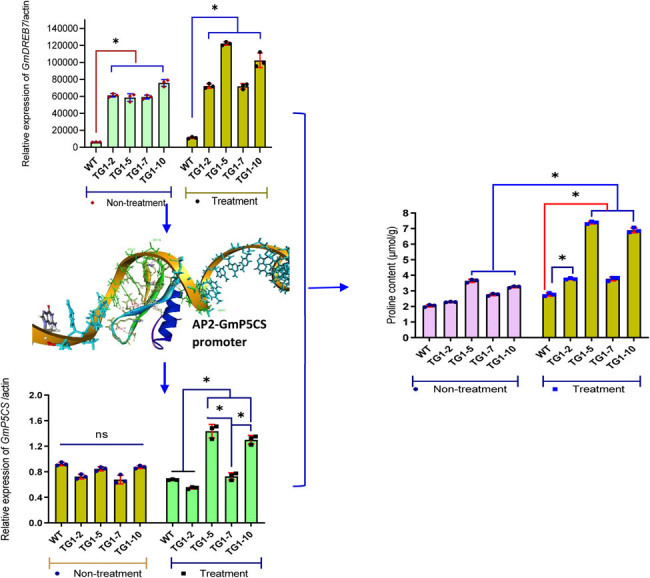

## 1 Introduction

Soybean [*Glycine max* (L.) Merr.] is an important food crop in many countries around the world. Soybean seeds provide protein and oil with high nutritional value. Soybean cultivation brings economic benefits and improves arable land [1,2]. However, in many areas, salinization of arable soil due to low precipitation, high evaporation rates, the use of chemical fertilizers, or rising sea levels has negatively impacted crops. Soil salinization is a serious problem that has a profoundly negative impact on agriculture. Excess salt adversely affects soil structure, fertility, crop growth, and yield, including soybean [3]. Soybeans respond to salt stress through the activity of some genes, including the dehydration response element binding (DREB) gene group, a group of transcription factors (TFs) that regulate downstream salt tolerance-related genes [4,5]. DREB proteins contain an AP2 domain of 58–60 amino acids. In the AP2 domain, 11 amino acids (binding DNA sites) bind to promoters of target genes in a downstream response to stress signals [6]. The TF DREB, a trans factor, regulates the expression of several genes responsive to drought and salt stress [7].

In soybeans, the *DREB* genes (*Glycine max DR – GmDREB*) can activate downstream genes in response to salt and drought stress [8]. Phang et al. [9] reported that the soybean genome has more than ten *DREB* genes: 73 *DREB* gene sequences have been identified in the *G. max* genome [4], and the results of phylogenetic analysis have formed six subgroups in the *DREB* genes in *G. max*. The *DREB* gene in soybeans has one exon and is unevenly distributed across the 19 chromosomes. At the same time, the authors identified 186 target genes in soybeans that play important roles in fructose and mannose metabolic pathways and were identified to be involved in the regulatory role of DREB proteins [4]. In the report analyzing *DREB* genes in wild soybean (*Glycine soja*) by Hou et al. [5], 56 DREB sequences of *Arabidopsis thaliana* were used as a query to search the *Glycine max* database, resulting in the identification of 103 *DREB* genes of the soybean genome. *DREB* genes are distributed on all soybean chromosomes. In the soybean DREB subfamily, the functions of several glycine max DREB (*GmDREB*) genes have been experimentally demonstrated. Overexpression of the *GmDREB2* gene enhanced drought and salt tolerance in transgenic plants [10,11,12,13].

The *GmDREB7* gene with ID 100101894, belonging to chromosome 20 of soybean, encodes dehydration-responsive element binding 7 (DREB7) protein, which was isolated and submitted to GenBank by Liu et al. [14]. Accordingly, the 624-bp *DREB7* mRNA encodes a protein of 207 amino acids. The AP2 domain of the DREB7 protein contains 11 amino acids (RGRSKERRWT) that are DNA-binding sites [14]. Proline is an osmolyte, and proline accumulation in plants is due to environmental stresses. Experimental evidence suggests a positive correlation between proline accumulation and stress tolerance. Delta-1-pyrroline-5-carboxylate synthase (P5CS), a key enzyme involved in proline synthesis from glutamate precursors, has been shown to play an essential role in proline accumulation in plants under drought stress [15]. Using genetic transformation and gene overexpression techniques, P5CS has been shown to confer desiccation tolerance by enhancing cellular proline content [16].

Therefore, several members of the *DREB* gene subfamily in the soybean genome have been identified playing roles in responses to abiotic stresses. However, many of these genes, including *GmDREB7*, remain insufficiently studied. Thus, the objective of this study was to investigate the regulatory role of the *GmDREB7* gene in modulating *GmP5CS* expression and proline accumulation in soybeans under salt stress through overexpression of the *GmDREB7* gene.

## 2 Materials and methods

### 2.1 Materials

The Legumes Research and Development Center, Field Crops Research Institute, Vietnam, provided DT26 soybean seeds. *Agrobacterium tumefaciens* AGL1 containing the expression construct pBI121_DREB7 is kept at the School of Biology, Thai Nguyen University of Education, Vietnam.

The nucleotide sequences of primer pairs used for PCR and real-time reverse transcription polymerase chain reaction (RT-PCR) analysis are shown in Table 1.

**Table 1:** Sequences of primer pairs used for RT-PCR and qRT-PCR

Primers	Sequence (5′−3′)	Primer annealing temperature (°C)
GmDR-F/MYC-R	ATGTTTTCCATCAATCATTTCTCC	58
	AAGTTCATCCTTCAGGTCCTC	58
qRT-DREB7-F/qRT-DREB7-R	TGCCGGAGTATCTGAGGAAC	61
	CTGAGATCAGCTTCTGCTCC	61
qRT-P5CSF/qRT-P5CSR	TGCTCGTGAGATGGCAGTTGC	60
	AGCCTGTTGAGCAGCAACCAC	60
qRT-ActNF/qRT-ActNR	GATCTTGCTGGTCGTGATCTT	60
	GTCTCCAACTCTTGCTCATAGTC	60

### 2.2 *Agrobacterium-*mediated transformation of soybean

Genetic transformation of the pBI121_GmDREB7 construct into soybean via the cotyledon node by *Agrobacterium,* and the generation of transgenic soybean plants performed according to Olhoft et al. [17] and Yang et al. [18]. Cotyledons were collected from germinated soybean seeds and soaked in *A. tumefaciens* AGL1 solution containing the vector pBI121_DREB7. After 30 min, the infected cotyledons were transferred to a co-culture medium. The transformed explants were washed with 500 mg L^−1^ cefotaxime for 10 min, then blotted dry and cultured in the shoot induction medium (SIM – first time) with 50 mg L^−1^ kanamycin for 15 days to regenerate multiple shoots. Next, the transformed explants were transferred to SIM (second time), and 500 mg L^−1^ cefotaxime and 75 mg L^−1^ kanamycin were added and cultured for 15 days. The selected surviving shoots were transferred to a shoot elongation medium, and 500 mg L^−1^ cefotaxime and 50 mg L^−1^ kanamycin were added for shoot elongation. The well-grown shoots were selected for transferring the rooting medium (RM), and 250 mg L^−1^ cefotaxime and 50 mg L^−1^ kanamycin were added to the medium for root regeneration and complete soybean plant formation. Transgenic soybean plants that grow well were transferred to grow on substrates in greenhouses. Soybean plants regenerated from *in vitro DREB7* gene-transformed explants grown on substrates are referred to as TG0 generation plants (TG0). Soybean plants germinated from seeds of TG0 plants are referred to as TG1 generation plants (TG1).

### 2.3 Confirmation of the insertion and expression of the *GmDREB7* into the transformed soybean genome

Confirmation of the insertion of the *GmDREB7* into the soybean genome: Total RNA was extracted from young leaves of transgenic and wild-type, non-transformed plants (WT) using a TrizolRIZOL kit, and cDNA was synthesized using a First Strand cDNA Synthesis Kit. The *GmDREB7* gene with primer pair GmDR-F/Cmyc-R was amplified using the PCR. The expected size of the cloned *GmDREB7* gene fragment is about 0.65 kb.

### 2.4 Analysis of transgenic soybean lines

Treatment of TG1 transgenic lines with salt stress: The seeds of TG0 plants germinated into TG1 plants and were grown in a greenhouse at an average temperature of 23°C (daytime) and 20°C (nighttime) with a photoperiod of 16 h. At the three-leaf stage (V3), TG1 and WT transgenic plants were treated with salt stress using NaCl. WT and TG1 transgenic plants were watered in the experimental group with 50 mL of NaCl (150 and 250 mM). The salinity treatment experiment was performed three times, with a 3-day interval between each trial. In the first treatment, the plants were watered with 150 mM NaCl solution, and in the second and third treatments, the plants were watered with 250 mM NaCl solution. In the control group, WT and TG1 transgenic plants were watered with 50 mL H_2_O/time three times, each time 3 days apart.

#### 2.4.1 Analysis of the transcription level of GmDREB7 in transgenic soybean plants and WT plants

The expression levels of *GmDREB7* and *GmP5CS* in transgenic soybean plants and WT plants in non-stressed and salt-stressed conditions were analyzed by real-time quantitative reverse transcription PCR (real-time qRT-PCR). The reference gene used in this analysis was SAc1 (GenBank: J01298.1). Real-time qRT-PCRs were performed in 20 μL, containing components such as primers, Master Mix, cDNA, and water. The thermal cycling of the real-time qRT-PCR consisted of 95°C (10 min), 40 cycles at 95°C (10 s), 58°C (10 s), and 72°C (20 s).

Real-time RT-PCR results were analyzed using Q-Rex version 1.0 (QIAGEN, Hilden, Germany). Transcription levels were calculated as *R* = 2^−∆∆*C*_t_^ [19].

#### 2.4.2 Proline content analysis in transgenic lines and WT plants

The proline amino acid content was determined and calculated according to Bates et al. [20] (μmol g^−1^ fresh weight).

### 2.5 Molecular docking analysis

#### 2.5.1 Creation of PDB files of proteins and DNA

To construct docking models, the promoter sequence of the downstream gene *GmP5CS* was designed to include *cis*-regulatory elements *DRE/CRT, ABRE*, and *GT-1* (Table 2), along with spacer regions to enhance structural stability. DNA models with varying bend angles in the β-DNA form were generated using Avogadro 1.2.0 [21]. The *GmDREB7* sequence (GenBank accession: NM_001248108.2; Gene ID: 100101894) and the AP2 domain of the DREB7 protein were retrieved (Table 2). The 3D structure of this domain, based on its amino acid sequence, was predicted using AlphaFold [22].

**Table 2:** Characterization and sequences of the AP2 regions, DNA binding sites, promoter of *GmDREB7*, and *cis-*acting motifs in the promoter

Regions/domain/motif	Sequences	Description
AP2	YRGVRRRDSGKWVCEVREPNKKSRIWLGTFPTAEMAARAHDVAAIALRGRSACLNFADS	The AP2 (APETALA2) domain of the DREB7 protein contains DNA binding sites
DNA binding sites	RGRRSKERRWT	DNA binding site (nucleotide-binding) includes 11 amino acids
Promoter of *GmP5CS*	TTACCGACAGCCGCCTGGTTAACGTGTAACTGGCCGACGAAAAACATGTGAGCCGCC	The sequence of *cis*-acting motifs of the promoter of the *GmP5CS* gene
DRE/CRT	GCCGAC	Abiotic stress-responsive: salt, cold, and drought
ABRE	ACGTG	ABA-responsive (ABA related to salt stress).
GT-1	GAAAAA/TGGTTA	Salt stress-responsive

*Molecular docking* between the AP2 domain and the target promoter was conducted using HADDOCK. The resulting complexes were visualized and analyzed in Chimera 1.19 [23]. The analysis focused on hydrogen bonding, electrostatic interactions, and the interaction interface. The key interacting amino acid residues and nucleotide bases were identified. Binding energy and docking scores were assessed to estimate the stability of the protein*–*DNA complex.

### 2.6 Statistical analyses

SPSS software for Windows version 29.0.2.0 (Armonk, NY, USA) (IBM Corp) [24] was used to process data using a one-way variance method. LSD and Duncan’s test were used to compare of differences at *α* = 0.05 level.

## 3 Results

### 3.1 Genetic transformation of the pBI121_GmDREB7 construct into soybean and creation of transgenic plants

**Figure 1:**
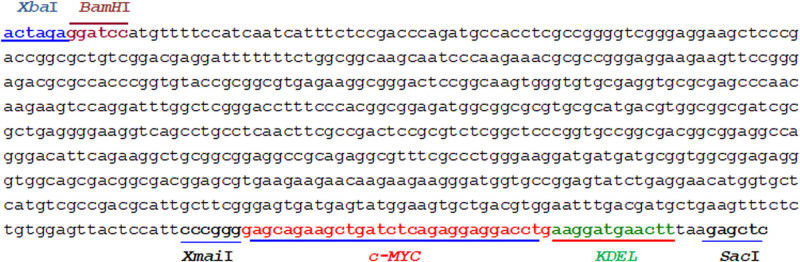
The designed and synthesized *GmDREB7* sequence contains the coding region, the *Xba*I and *BamH*I cutting sites at the 5′-end, and the *Sac*I at the 3′-end.

The pBI121_GmDREB7 construct was designed with the CaMV35S promoter, the coding region of the *GmDREB7*, and the sequences containing the cutting sites of the restriction enzymes. The *GmDREB7* gene was synthesized based on the information from the Gm*DREB7* sequence of soybean with the accession number on GenBank NM_001248108.2. The *GmDREB7* gene includes a coding region of 624 bp, the c-MYC antigen coding sequence (33 bp), the KDEL fragment (12 bp), and the restriction enzyme cutting sites *Xba*I, *BamH*I, *Xma*I, and *Sac*I (Figure 1).

**Figure 2:**
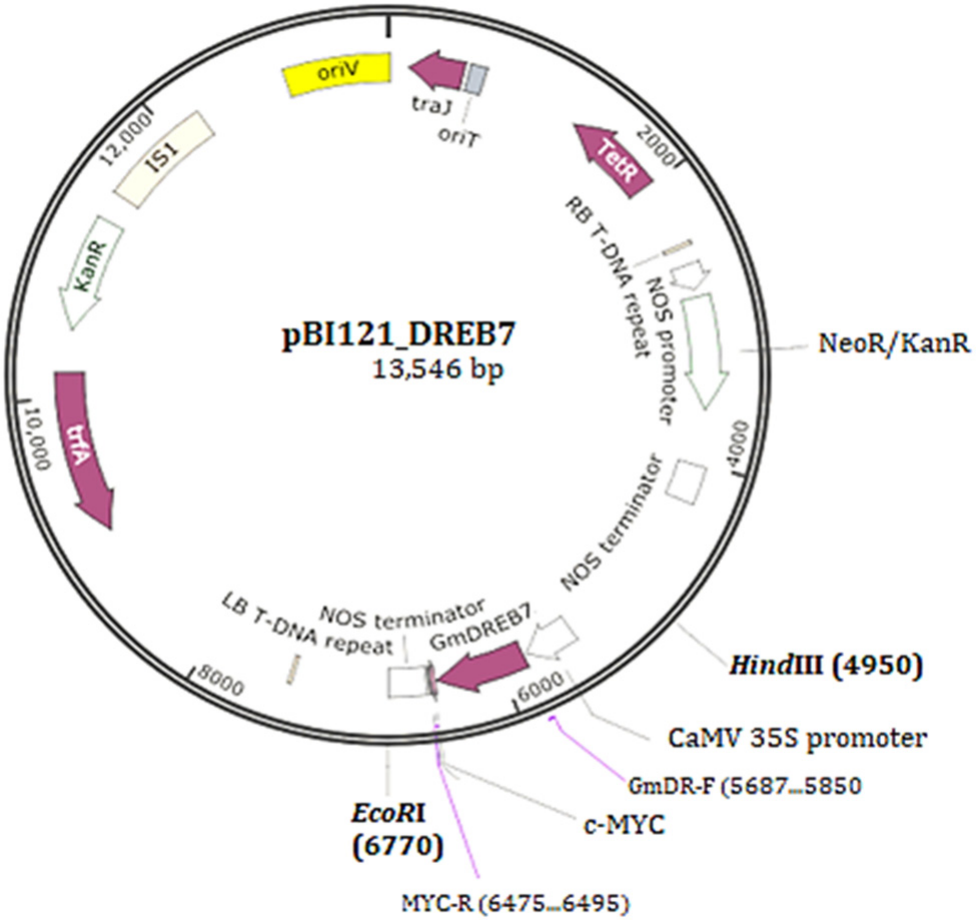
Schematic diagram of the pBI121_GmDREB7 construct. LB: left T-DNA border; RB: right T-DNA border; CaMV35S: Cauliflower mosaic virus 35S promoter; *GmDREB7*: coding region of the *GmDREB7*gene; Noster: transcription terminator sequence; *Hind* III and *EcoR*I: cleavage sites of these restriction enzymes; GmDR-F: forward primer, MYC-R: reverse primer and primer pair GmDR-F/MYC-R was used to amplify the *GmDREB7* transgene.

The pBI121_DREB7 vector contains the *KanR* gene, encoding the neomycin-phospho-transferase II (nptII) (Figure 2).

DT26 soybean seeds were germinated on a murashige and Skoog medium (MS), and cotyledons were collected to use as the material to receive the gene. The pBI121_GmDREB7 construct was transformed into a DT26 soybean cultivar through the cotyledon axils by infection with *A. tumefaciens* (Figure S1). From 90 transformed cotyledon fragments, *in vitro* regeneration and kanamycin selection resulted in 18 healthy shoots, which were transferred to the RM. Ten plants in the TG0 transgenic generation (TG) that grew well were selected and planted on substrates in a greenhouse. In the control group, 20 cotyledons were not transformed, shoots were regenerated, and non-transformation soybean plants (wild type: WT plants) were created. These plants were grown in an environment without the use of selective antibiotics. Eight plants that grew well were selected and planted on substrates in a greenhouse.

### 3.2 Results of the analysis of transgenic soybean plants

The results of the analysis of ten plants transformed with the *GmDREB7* gene in the TG0 generation and WT plants by RT-PCR with the primers GmDR-F/MYC-R are shown in Figure S2. The electrophoresis image of the RT-PCR products shows that nine TG0 plants are positive for RT-PCR (TG0-1, TG0-2, TG0-3, TG0-4, TG0-5, TG0-6, TG0-7, TG0-9, and TG0-10) with a DNA band of about 0.65 kb in size, as theoretically calculated. TG0-9 plants and WT plants do not have a DNA band. This result confirmed that the construct carrying the *GmDREB7* gene has integrated into the genome of nine TG0 transgenic soybean plants and shows transcriptional activity.

Monitoring the growth and development of nine transgenic plants in the TG0 generation with positive RT-PCR results showed that seven plants flowered and produced fruits (TG0-2, TG0-3, TG0-4, TG0-5, TG0-6, TG0-7, TG0-10). However, only the fruits of four TG0 plants, TG0-2, TG0-5, TG0-7, and TG0-10, produced seeds; the remaining plants, TG0-3, TG0-4, and TG0-6, produced empty fruits and did not produce seeds. The seeds of four TG0 plants (TG0-2, TG0-5, TG0-7, and TG0-10) were germinated and grown in a greenhouse to produce four TG1 transgenic lines, namely TG1-2, TG1-5, TG1-7, and TG1-10. Salt stress treatment of TG1 transgenic lines and WT plants with NaCl from 150 to 250 mM yielded, with the first irrigation, 50 mL of 150 mM NaCl, and the second and third irrigations, 50 mL of 250 mM NaCl (Figure S3).

**Figure 3:**
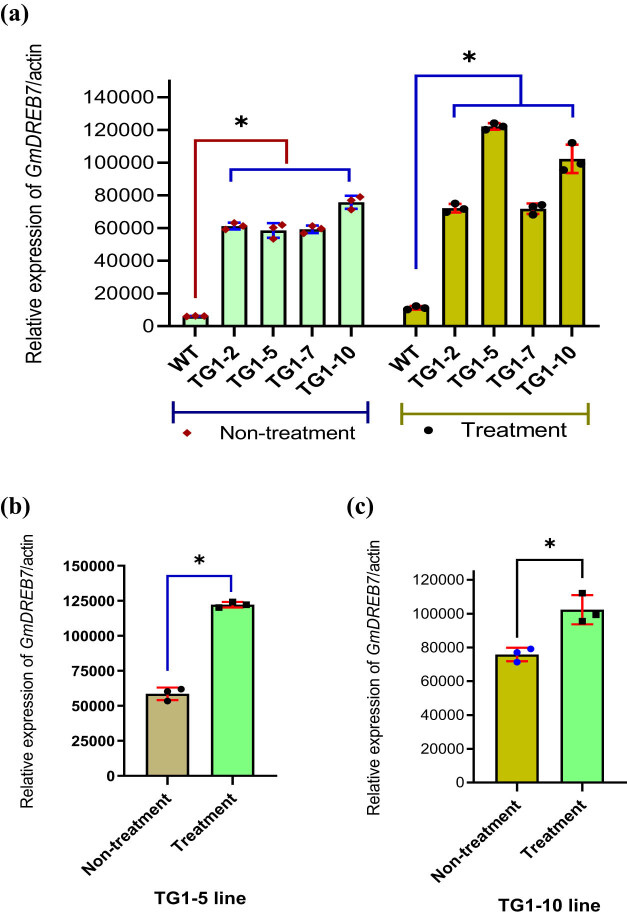
The diagram compares the transcription levels of the *GmDREB7* gene by real-time RT-PCR in WT and TG1 generation transgenic soybean lines under untreated and salt-treated conditions, using 50 mL of 250 mM NaCl after 2 days of the second treatment. (a) The graph shows the transcription levels of *GmDREB7* in transgenic soybean lines compared to WT plants under non-treatment and salt stress treatment (*P* < 0.05). (b) and (c) Comparison chart of transcription levels of *GmDREB7* in TG1-5 and TG1-10 transgenic soybean lines between salt stress treatment and untreated conditions (*P* < 0.05). The reference gene used in real-time RT-PCR analysis was *SAc1* (Actin); WT: wild-type, non-transgenic plants; TG1-2, TG1-5, TG1-7, and TG1-10: transgenic lines in TG1 generation. The * symbols above the columns of the graph represent statistically significant differences at *P* < 0.05. Bars display geometric means, and error bars represent geometric standard deviation (SD). Dots represent individual samples.

Observing the morphology of the plants after 2 days of each salinity treatment showed that, in the first treatment, the morphology of WT plants and transgenic lines did not show any morphological expression due to the impact of 150 mM NaCl (Figure S3a). However, the second treatment (Figure S3b) with 250 mM NaCl affected the morphology of transgenic soybean plants and WT plants, and the most substantial effect was after the third treatment (Figure S3c). In Figure 3c, WT plants and TG1-7 soybean lines showed the most evident signs of water loss due to salinity stress.

WT plants and TG1 transgenic soybean lines after 2 days of the second treatment with 50 mL of 250 mM NaCl (Figure S3b) were selected to analyze the *GmDREB7* gene expression level of the transgenic lines and WT by real-time RT-PCR. The leaves of WT and transgenic soybeans in the TG1 generation were used to extract total RNA, generate cDNA, and analyze the transcription level of the *GmDREB7* of WT plants and four transgenic lines by real-time RT-PCR with the primer pair qRT-DREB7-F/qRT-DREB7-R; the results are shown in Figure 3. In Figure 3, all transgenic soybean lines exhibited higher transcription levels of the *GmDREB7* gene compared to WT plants. Under salt stress conditions, after salinity treatment with 250 mM NaCl, the transgenic soybean lines TG1-5 and TG1-10 had the strongest and highest transcription levels compared to those under non-salinity treatment conditions (*P* < 0.05).

**Figure 4:**
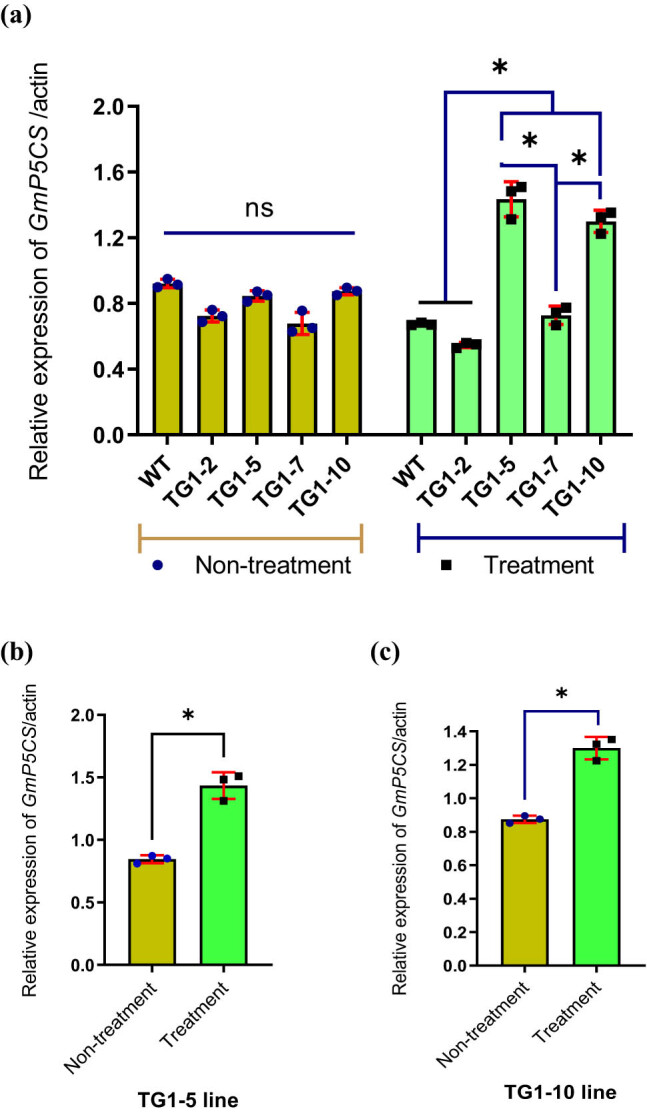
The diagram compares *GmP5CS* gene transcription levels in four TG1 generation transgenic soybean lines and wild-type plants using real-time RT-PCR. (a) The graph shows the transcription levels of *GmP5CS* in transgenic soybean lines compared to WT plants under non-treatment and salt stress treatment (*P* < 0.05). (b) and (c) Comparison chart of transcription levels of *GmP5CS* in TG1-5 and TG1-10 transgenic soybean lines between salt stress treatment and untreated conditions (*P* < 0.05). The reference gene used in real-time RT-PCR analysis was *SAc1* (Actin); WT: wild-type, non-transgenic plants; TG1-2, TG1-5, TG1-7, and TG1-10: transgenic lines in TG1 generation. The * symbols above the columns of the graph represent statistically significant differences at *P* < 0.05. Bars display geometric means, and error bars represent geometric standard deviation (SD). Dots represent individual samples.

The amino acid proline is an osmolyte; increasing the proline content will increase the water retention capacity of the cell, and the plant will be resistant to salt stress. Therefore, this study continues to investigate the relationship between the overexpression of the *GmDREB7* gene and the transcription of the gene encoding the key enzyme P5CS, an enzyme involved in proline synthesis, in transgenic lines and WT plants under salt stress and normal conditions. The results of analyzing the transcription level of the *GmP5CS* gene of the *GmDREB7* transgenic lines and WT plants showed that the two transgenic lines, TG1-5 and TG1-10, had higher expression levels than WT plants and higher than in non-salt-treated conditions with *P* < 0.05 (Figure 4).

**Figure 5:**
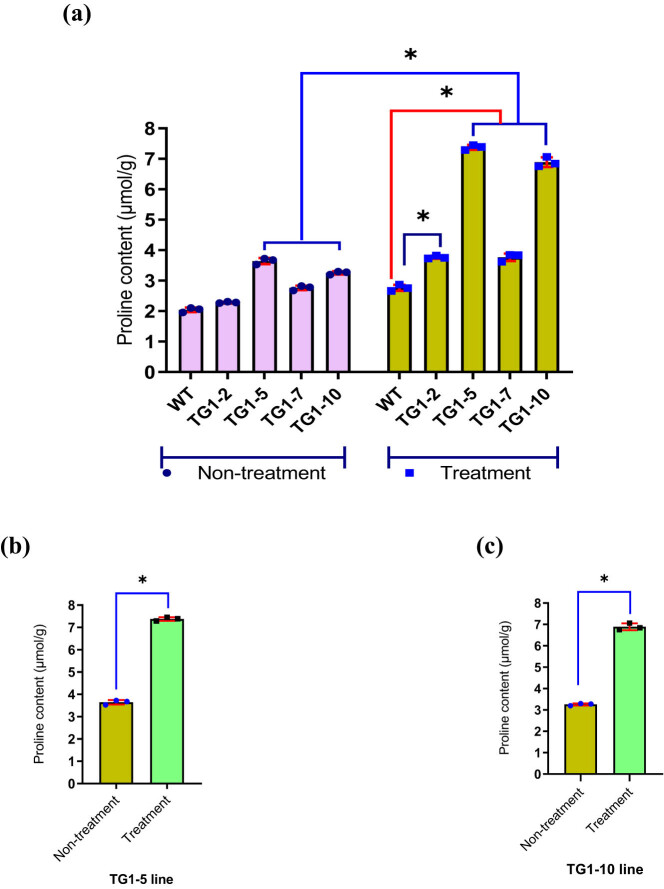
The diagram compares the proline content of the TG1 generation transgenic soybean lines with that of the WT plants. (a) The graph shows the proline content of transgenic soybean lines compared to WT plants under non-treatment and salt stress treatment (*P* < 0.05). (b) and (c) Comparison chart of proline content in TG1-5 and TG1-10 transgenic soybean lines between salt stress treatment and untreated conditions (*P* < 0.05). WT: wild-type, non-transgenic plants; TG1-2, TG1-5, TG1-7, and TG1-10: transgenic lines in TG1 generation. The * symbols above the columns of the graph represent statistically significant differences at *P* < 0.05. Bars display geometric means, and error bars represent geometric standard deviation (SD). Dots represent individual samples.

The proline content of four transgenic soybean lines, TG1-2, TG1-5, TG1-7, TG1-10, and WT plants under untreated and salt stress conditions with 50 mL of 250 mM NaCl was analyzed, and the results are presented in Figure 5.

The results in Figure 5 showed that the *GmDREB7* transgenic lines and WT plants had higher proline content than untreated conditions (Figure 5a) and increased from 135.29 to 211.35% (Table S1). The proline content in transgenic lines was higher than in WT plants and increased from 136.59 to 267.03%. Among the transgenic soybean lines, lines TG1-5 and TG1-10 had the highest proline contents, 7.37 ± 0.05 (μmol/g) and 6.89 ± 0.09 (μmol/g), respectively (*P* < 0.05) (Table S1), and the proline content of the two transgenic lines under salt stress conditions was both higher than under untreated conditions (Figure 5b and c). These results suggested that the *GmDREB7* transgenic soybean lines may have had higher salt tolerance than WT plants, and lines TG1-5 and TG1-10 had the highest salt tolerance among the transgenic soybean lines in this study.

### 3.3 Docking between the AP2 region of the DREB7 protein and the promoter regions of the *GmP5CS*

**Figure 6:**
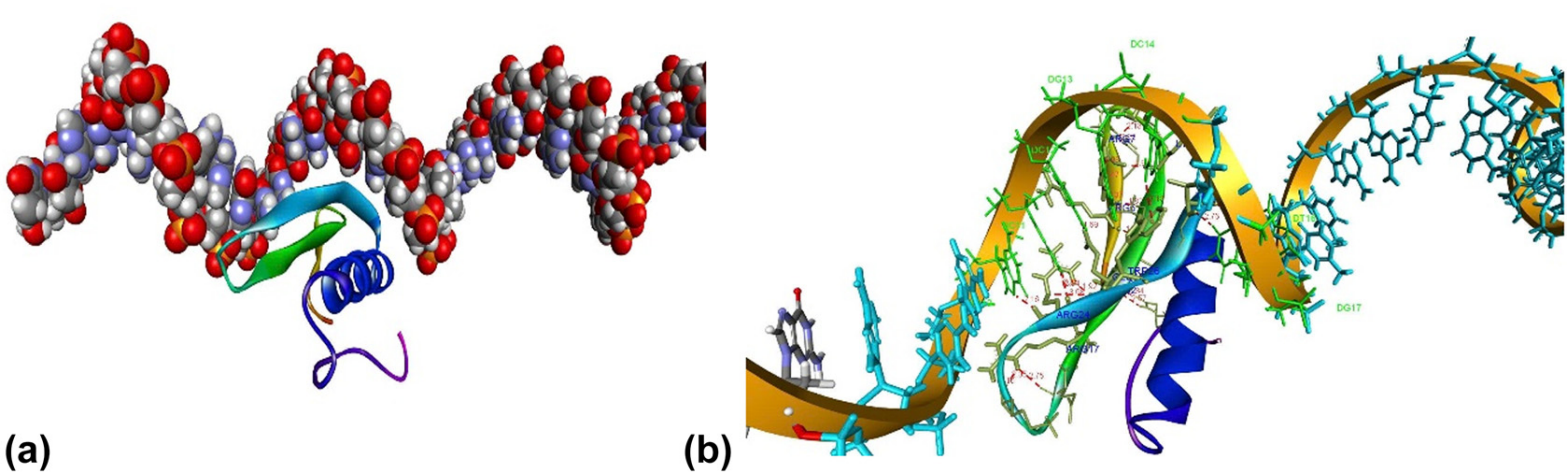
Docking model of the AP2 domain and its hydrogen bonding interactions with the target DNA (GmP5CS promoter). (a) The top-ranked docking model, selected based on the highest HADDOCK score, highlights conserved AP2 motifs involved in DNA binding. (b) Hydrogen bond network between AP2 amino acid residues and the major and minor grooves of the target DNA. The α-helix and β-sheet regions of AP2 are shown in royal blue and light blue, respectively, while the DNA double helix is colored orange.

To confirm the interaction between the AP2 domain of the DREB7 protein and the promoter of the *GmP5CS* gene, an *in silico* molecular docking analysis was conducted. The AP2 region of DREB7 comprises 59 amino acids, including an 11-amino-acid DNA-binding domain. The GmP5CS promoter contains *DRE/CRT, ABRE*, and *GT-1* motifs, which are associated with responses to abiotic stresses such as drought and salinity (Table 2). The predicted interaction model between the AP2 domain and the GmP5CS promoter is illustrated in Figure 6.

Hydrogen bonds (H-bonds) are critical for the affinity and specificity of the AP2*–*DNA interaction. As shown in Figure 6b, the DNA-binding residues of the AP2 domain, including ARG2, GLY3, ARG5, ARG7, TRP9, LYS11, GLU15, ARG17, ARG24, TRP26, and THR29, form H-bonds with the promoter region containing *cis*-acting elements at DRE/CRT and GT-1 motifs, including DG10, DC11, DC12, DG13, DC14, DT16, and DG17. These findings confirm the binding of the DREB7 AP2 domain to the GmP5CS promoter. Detailed docking parameters are provided in Table 3.

**Table 3:** Docking scores and binding energy parameters for AP2–GmP5CS promoter complexes

Docking properties	AP2–GmP5CS promoter complex
Binding score (kcal/mol)	−83.8 ± −6.2
RMSD from the overall lowest-energy structure	6.0 ± 0.1
van der Waals energy	−35.5 ± 9.6
Electrostatic energy	−331.6 ± −24.4
Desolvation energy	11.2 ± 1.0
Restraints violation energy	68.1 ± 25.9
Buried surface area	1267.2 ± 71.6
*Z*-score	−2.2

Table 3 presents docking parameters for the AP2–GmP5CS promoter complex, highlighting markedly negative binding energy (−83.8 ± 6.2 kcal/mol), which reflects strong binding affinity between the AP2 domain of the DREB7 protein and the GmP5CS promoter. The relatively small standard deviation (±6.2 kcal/mol) suggests consistent binding energy across docking conformations. The van der Waals energy (−35.5 ± 9.6 kcal/mol) indicates favorable intermolecular interactions that contribute to complex stability. A large buried surface area (1267.2 ± 71.6 Å²) suggests extensive molecular contact between AP2 and the DNA, supporting tight binding. The root mean square deviation (RMSD) of 6.0 ± 0.1 Å reflects proximity to the lowest-energy conformation, with the low standard deviation (±0.1 Å), indicating high structural consistency among docking models. The negative *Z*-score (−2.2) suggests that the binding energy is significantly lower than that of randomly generated complexes, confirming the specificity and statistical significance of the interaction. These results confirmed the specific interaction between the DREB7 protein and the GmP5CS promoter based on *in silico* molecular docking analysis.

## 4 Discussion

Abiotic stress conditions adversely affect plants, and DREB TFs are essential regulators of this stress response [25,26,27,28,29]. Plants, including soybeans, always face adverse abiotic impacts such as drought and saline soil, and soybeans’ positive response is reflected in increased gene expression when environmental stress signals occur. Salt stress is recognized as one of the most detrimental factors affecting crop productivity, primarily due to its induction of both osmotic imbalance and ion toxicity. Soybean exhibits moderate sensitivity to salinity, with yield reductions reaching up to 40%, depending on salt concentration levels [30]. In a comparative evaluation of over 20 soybean cultivars, an increase in salinity from 2 to 7 dS/m resulted in an approximate 40% decline in yield. Field-based assessments further revealed that salt-sensitive genotypes experienced up to 37% greater yield loss compared to salt-tolerant counterparts under similar saline conditions [31]. Additionally, in the “Williams” variety, salinity stress was found to reduce seed protein and oil content by as much as 39% [30].

Functional and regulatory genes involved in abiotic stresses, major abiotic stress signals, and plant signaling pathways were investigated [32]. Transcriptome, sRNAome, degradome, and expression analysis of TF genes involved in plant responses to abiotic stresses are also interesting [33,34]. DREB protein is a TF that has been identified to have the ability to activate and enhance the transcription of downstream genes [4,5]. The *DREB1, DREB2,* and *DREB6* genes of soybeans are closely related to the response to cold, drought, and salinity stress [11,12,35]. Overexpression of *DREB1* in soybeans has resulted in drought tolerance, enhanced root system, and delayed senescence of transgenic leaves [36]. Upon receiving external abiotic stress signals, the transcriptional activity of the *DREB2* and *DREB6* genes is enhanced, resulting in increased proline accumulation and enhanced drought and salt tolerance in transgenic soybean plants [11,12,13]. In a previous study, the expression of the soybean *DREB7* gene was analyzed to determine its association with two genes, *RD29A* and *SODFe*, in *DREB7* transgenic tobacco plants under both normal (untreated) and salt stress conditions. In the previous study, the expression of the soybean *DREB7* gene was analyzed, determining the association with two genes, *RD29A* and *SODFe*, in *DREB7* transgenic tobacco plants under normal (untreated) and salt stress conditions. Overexpression of the soybean *GmDREB7* under salt stress conditions reduced the transcription levels of the two tobacco genes *RD29A* and *SODFe* [37]. In this study, the function of the *GmDREB7* gene continues to be investigated in soybean plants in non-stressed and salt-stressed environments. Overexpression of the *GmDREB7* gene in soybean plants was demonstrated by increased mRNA transcription, as evidenced by quantitative real-time reverse transcription polymerase chain reaction (qRT-PCR) values in transgenic lines, which were higher than those in WT plants under both untreated and salt stress conditions with 250 mM NaCl. When there was a salt stress signal, the response of soybean plants was shown by the higher transcription levels of transgenic lines than WT plants, and the *GmDREB7* gene was transcribed most strongly in the two lines TG1-5 and TG1-10, with *P* < 0.5 (Figure 4). This result was also found in some reports analyzing the transcriptional expression levels of the *GmDREB2* and *GmDREB6* genes under drought and salt stress conditions [11,12,13].

Proline plays various roles in plant growth, development, and physiology. It is synthesized in adaptive responses to external stresses. Proline, a critical osmolyte and antioxidant, plays a vital role in regulating abiotic stress tolerance in plants, especially its key biosynthetic enzyme, P5CS, which is always positively responsive to drought and salinity stress. High proline plant accumulation is often correlated with abiotic stress factors such as salinity and drought [38,39].

Under salt stress conditions, proline accumulation increases in the cytoplasm and participates in osmotic adjustment, thereby enhancing the cells’ water retention ability and increasing the plant’s tolerance to drought and salt [15,40]. In this study, the expression of the gene encoding the enzyme P5CS under salt stress signals was most evident in the two lines TG1-5 and TG1-10 (*P* < 0.05) (Figure 5). Under salt stress conditions, the increase in proline accumulation, caused by the increased transcriptional expression of the *GmP5CS* in soybean, as determined, was increased from 135.29 to 211.35% compared to the non-salt stress conditions. The proline content in the *DREB7* transgenic lines increased from 136.59 to 267.03% compared to the WT plants (Table S1). Several studies on potato, common bean, and soybean have also experimentally demonstrated the association between GmP5CS gene transcriptional enhancement and proline content [11,12,41,42,43]. In this study, it can be hypothesized that the TF DREB7 in soybean, through its conserved DNA-binding sequence RGRRSKERRWT within the AP2/ERF domain, is capable of interacting with the GCC motif in the promoter region of the *GmP5CS* gene, a gene essential in the proline biosynthesis pathway under stress conditions.

The amino acids in the sequence RGRRSKERRWT, particularly the positively charged residues such as arginine (R) and lysine (K), are characteristic positions for interaction with the minor groove of DNA. These residues have been shown to play a role in target DNA recognition by various TFs in the ERF family. Although DREB is primarily known for recognizing the DRE/CRT motif (A/GCCGAC), recent studies suggest an expanded binding capability with the GCC box motif in the promoter context of stress-responsive genes, especially in leguminous plants. Previous studies have reported that the promoter sequence of *GmP5CS* contains *cis-*regulatory motifs, such as the GT-1 (256, 21243, 1641) and GCC (21899) [12,44]. Therefore, the hypothesis that can be posed is that DREB7 may bind to the GCC box in the promoter region of P5CS, thereby regulating the transcription of this gene under adverse environmental conditions.

This hypothesis can be tested through experimental methods such as EMSA, yeast one-hybrid assay, or molecular docking simulations between the AP2 domain and the GCC sequence. Confirming this interaction would further elucidate the regulatory role of DREB7 in the stress tolerance mechanism in soybeans. Thus, the results of the analysis of the relationship between the overexpression of the *GmDREB7* gene in TG1 transgenic soybean lines and the increase in the transcription level of the *GmP5CS* gene and the increase in the accumulation of proline amino acid under salt stress conditions showed that the activity of *GmDREB7* gene produces DREB7 protein, which can activate the transcription of *GmP5CS* gene and can increase the accumulation of proline (osmotic substance) and increase the water retention capacity of cells, enhancing the physiological drought and salt tolerance of transgenic soybean plants. The *GmDREB7* gene may be a potential candidate for improving the salt resistance of soybean plants; however, further analysis is necessary to confirm this conclusion.

Previous studies have explored protein–DNA interactions using molecular docking, including investigations of the GCC-box binding domain in complexes with DNA [45,46,47]. While the AP2 domains of DREB TFs are known to contain residues that interact with *cis*-regulatory motifs in target gene promoters, limited research has addressed docking models that explain the transcriptional activation function of soybean DREB7 in stress-responsive gene regulation. In this study, key amino acid residues of the AP2 domain-ARG2, GLY3, ARG5, ARG7, TRP9, LYS11, GLU15, ARG17, ARG24, TRP26, and THR29-formed hydrogen bonds with *cis*-acting elements in the *GmP5CS* promoter (Figure 6b). These elements, located in the terminal promoter region, include GT-1 (TGGTTA), GCC, and ACGTG motifs, which are associated with drought and salt stress responses. The molecular docking results (Table 3) support the observed upregulation of *GmP5CS* expression in transgenic soybeans overexpressing *GmDREB7*. However, further experimental validation is necessary to confirm the proposed docking model and its functional relevance.

*Agrobacterium*-mediated gene transfer into soybean was first reported in 1988 [48,49], utilizing *A. tumefaciens* infection through wounds in the cotyledonary node. This technique has since become widely adopted due to its high efficiency. To date, numerous genetically modified (GM) soybean cultivars have been developed, representing the most significant proportion of GM crops worldwide and serving various applications in food, nutrition, industry, and pharmaceuticals [50]. Beyond varietal development, genetic transformation has also proven to be a valuable tool for functional genomics research [51].

In the present study, we reintroduced the gene encoding the soybean TF DREB7 into soybean plants to investigate whether its overexpression could activate downstream genes involved in salt stress responses. The results demonstrated that *GmDREB7* overexpression significantly enhanced the transcription of the *GmP5CS* gene, thereby improving the plant’s tolerance to salinity. Molecular docking and interaction analyses further confirmed a direct association between the DREB7 TF and the promoter region of the *GmP5CS* gene.

Historically, conventional soybean breeding methods – such as population selection, sexual hybridization, and induced mutagenesis – have contributed substantially to varietal improvement; however, they often require extensive time and labor. Modern breeding strategies, based on genetic engineering and the integration of transgenic approaches with traditional hybridization, are now favored for their superior efficiency, precision, and speed. These approaches also preserve genetic diversity and enhance adaptability to environmental stressors. Based on our findings, the transgenic lines TG1-5 and TG1-10 exhibited significantly enhanced salt tolerance and are promising candidates for incorporation into breeding programs aimed at developing soybean varieties suited for saline environments, including areas affected by seawater intrusion and soil salinization from excessive chemical fertilizer use.

This study provides functional evidence that DREB7 acts as a positive regulator of salt tolerance in soybeans by directly activating *GmP5CS* transcription and promoting proline accumulation. It is the first to demonstrate this relationship through both molecular and computational approaches. The validated transgenic lines TG1-5 and TG1-10 are promising candidates for the development of salt-tolerant soybean cultivars through genetic engineering or molecular breeding strategies.

## 5 Conclusions

In conclusion, the successful transformation of soybean with the pBI121_DREB7 construct-harboring the CaMV35S promoter and the *GmDREB7* gene-resulted in the TG1 generation of four transgenic lines. The *GmDREB7* gene was effectively overexpressed in both control and NaCl-treated conditions. Among the generated lines, TG1-5 and TG1-10 exhibited significantly higher DREB7 transcript levels (*P* < 0.05). Under salt stress, these two lines also showed markedly elevated expression of the *GmP5CS* gene and increased proline accumulation (*P* < 0.05). The interaction between the DREB7 protein and the GmP5CS promoter was specifically identified through *in silico* molecular docking analysis. These findings indicate that *GmDREB7* may contribute to enhancing *GmP5CS* transcription and proline biosynthesis under saline conditions. Therefore, *GmDREB7* can be a promising candidate gene for improving salt tolerance in soybeans.

## Supplementary Material

Supplementary material
