# Supplementary material for: The DREB7 transcription factor enhances salt tolerance in soybean plants under salt stress
Source: Open Life Sci. 2025 Aug 20;20(1):20251153. doi: 10.1515/biol-2025-1153 (PMC12412372; doi:10.1515/biol-2025-1153)
Supplement: Supplementary material [file biol-2025-1153-sm.pdf]

# Supplementary material

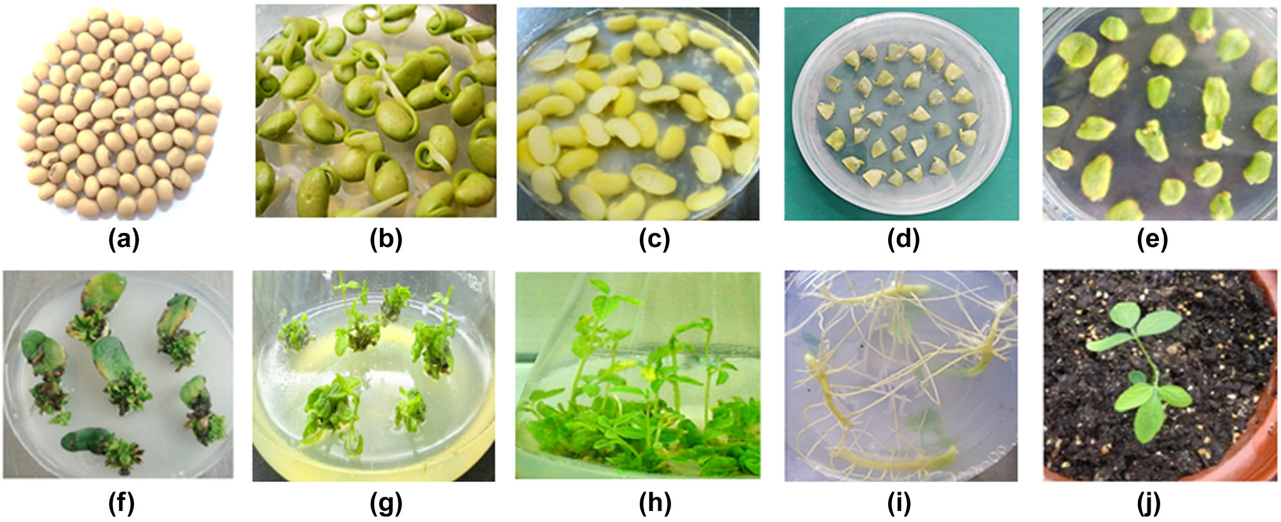


**Figure S1:** Transformation of pBI121_DREB7 construct and regeneration of transgenic soybeans. (a) DT26 soybean seeds were sterilized with chlorine gas; (b) Seeds were germinated on GM to create transformation material; (c) Soaking cotyledons in A. tumefaciens solution; (d) Cotyledon fragments infected with A. tumefaciens were cultured on CCM; (e)–(h) Shoot induction and selection on SIM 1 and SIM 2; (i) Selection for shoot elongation on SEM; (k) Rooting; (m) Transgenic soybean plants grown on substrate.

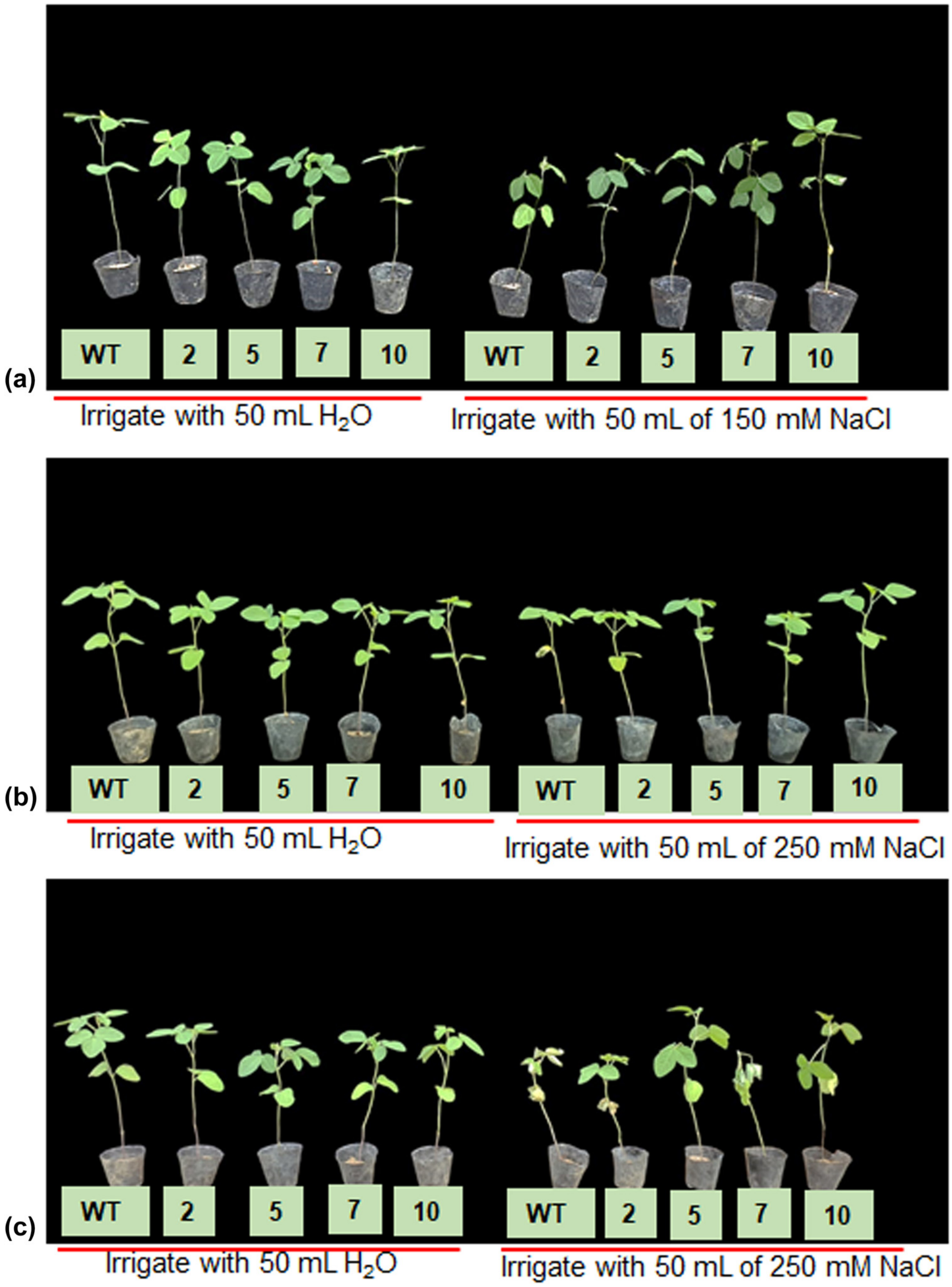


**Figure S2:** Images of transgenic soybean plants in TG1 generation and WT plants under watering conditions and salt stress treatment by NaCl. (a) Salt stress treatment with 150 mM NaCl after 3 days without watering; (b) Salt stress treatment with 250 mM NaCl after the next 2 days; (c) Salt stress treatment with 250 mM NaCl after the next 3 days. WT: Wild-type, non-transgenic plants; 2, 5, 7, and 10: Transgenic soybean lines in TG1 generation, TG1-2, TG-5, TG1-7, and TG1-10.

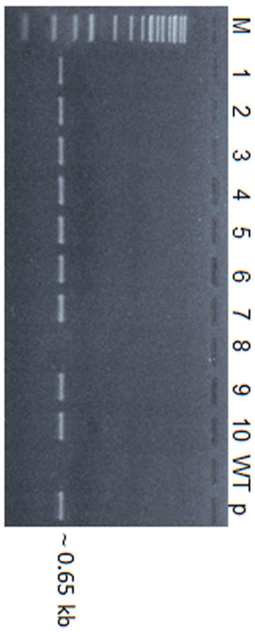


**Figure S3:** Electrophoresis images of RT-PCR products from transformed soybean plants in TG0 and WT plants. M: 1 kb DNA ladder, 1-10: TG0 transgenic plants; WT: non-transformed plants; p: plasmid pBI121_DREB7.

**Table S1:** Proline content of transgenic soybean lines in G1 generation and WT plants after two days of the second treatment with 250 mM NaCl

| Non-transgenic plants and transgenic soybean lines | Proline content in well-watered condition (µmol/g) | Proline content at salinity treatment with 250 mM NaCl (µmol/g) | Increased rate of proline accumulation under salt stress conditions compared to well-watered conditions (%) | Increased rate of proline accumulation in transgenic lines compared to non-transgenic plants (%) |
|---|---|---|---|---|
| WT | $2.04^{a} \pm 0.04$ | $2.76^{a} \pm 0.06$ | 135.29 | 100 |
| G1-2 | $2.29^{b} \pm 0.01$ | $3.77^{b} \pm 0.03$ | 164.63 | 136.59 |
| G1-5 | $3.64^{e} \pm 0.06$ | $7.37^{d} \pm 0.05$ | 202.47 | 267.03 |
| G1-7 | $2.76^{c} \pm 0.04$ | $3.83^{b} \pm 0.01$ | 138.77 | 138.77 |
| G1-10 | $3.26^{d} \pm 0.03$ | $6.89^{c} \pm 0.09$ | 211.35 | 249.64 |

*Note*: WT: Wild-type, non-transgenic plants; Different letters in the same column indicate differences at $P < 0.05$ ($n = 3$); The symbol ± represents standard error.
